# YT93-159: A High-Yield and Early-Maturing Sugarcane Variety with Superior Agronomic Traits

**DOI:** 10.3390/plants15213464

**Published:** 2026-11-06

**Authors:** Zilin Wu, Xueting Li, Rui Liu, Qiaoying Zeng, Fangyin Pan, Nannan Zhang, Jiayun Wu, Qinnan Wang

**Affiliations:** 1 Guangdong Sugarcane Genetic Improvement Engineering Centre, Institute of Nanfan & Seed Industry, Guangdong Academy of Sciences, Guangzhou 510316, China; wuzilin2008@126.com (Z.W.); lxt19910226@163.com (X.L.); liurui701@126.com (R.L.); zengqiaoying123@163.com (Q.Z.); zhangnn23@mail2.sysu.edu.cn (N.Z.); 2 Zhanjiang Research Center, Institute of Nanfan & Seed Industry, Guangdong Academy of Sciences, Zhanjiang 524300, China; panfy12@163.com

**Keywords:** sugarcane, YT93-159, early maturity, high sugar content, stress tolerance, molecular regulation

## Abstract

Sugarcane is an essential global industrial cash crop for sugar and bioenergy production, and breeding superior varieties is critical to improving industrial benefits and adapting to changing climatic environments. This review synthesizes the published breeding history, agronomic trial records, juice-quality evaluations, and transcriptomic analyses of the elite sugarcane variety YT93-159, and uses this variety as a model case to discuss the achievements and challenges of sugarcane breeding in South China. YT93-159 was bred through artificial hybridization using YN73-204 as the female parent and CP72-1210 as the male parent. Data compiled from variety-registration archives, peer-reviewed studies, and the authors’ previously published transcriptomic analyses indicate that YT93-159 integrates excellent parental advantages, with prominent early sugar accumulation, high yield, stable stress resistance, and wide adaptability. On the basis of differentially expressed genes identified in published transcriptomic datasets, its superior comprehensive traits may be co-regulated by candidate pathways including carbon fixation, sucrose metabolism, and plant hormone signal transduction; however, several pathway members showed the opposite expression changes, and these mechanisms should be regarded as hypotheses requiring further functional validation. This variety has achieved large-scale popularization in China and possesses excellent functional nutritional values. By clarifying the phenotypic and proposed molecular basis of YT93-159′s superior traits, this review provides a valuable germplasm resource profile and theoretical reference for sugarcane genetic improvement and sustainable industrial development.

## 1. Introduction

Sugarcane (*Saccharum* spp.) is an indispensable economic crop in tropical and subtropical agricultural systems, contributing to more than 80% of global commercial sugar output and emerging as a critical raw material for bioethanol power generation and clean energy production [1]. In China, sugarcane cultivation is mainly concentrated in Guangxi, Yunnan, Guangdong and other southern provinces, forming a characteristic pillar industry that drives regional farmers’ income growth and rural economic development. However, with the frequent occurrence of extreme high temperature, drought, and typhoon weather in recent years, coupled with the continuous variation of sugarcane pathogens, most sugarcane varieties generally face prominent problems such as late maturity, low sugar accumulation efficiency, unstable yield, and weak comprehensive stress resistance, which restrict the sustainable development of the sugarcane industry [2,3]. Therefore, breeding and popularizing new sugarcane varieties with early maturity, high yield, high sugar content, and strong multi-stress adaptability has become a core goal of current sugarcane genetic improvement [4,5].

Yuetang 93159 (YT93-159) is a representative elite sugarcane variety independently bred in South China, with excellent comprehensive agronomic performance. Phenotypically, YT93-159 has strong tillering ability, rapid canopy closure, early and efficient sugar accumulation, excellent resistance to biotic and abiotic stresses, coupled with low post-harvest sucrose inversion rate and superior storage tolerance, showing prominent application advantages in large-scale cultivation (unpublished official variety-registration document). With the rapid development of omics technology, transcriptome sequencing has become a mainstream technical means to analyze the molecular regulatory mechanism of excellent agronomic traits in sugarcane, and can effectively reveal the key functional genes and core metabolic pathways regulating yield formation, sugar quality improvement and stress tolerance [6,7]. At present, the molecular mechanisms of mainstream sugarcane varieties such as Guitang 42 (GT42), Guitang 44 (GT44), Liucheng 05-136 (LC05-136) and Yunzhe 08-1609 (YZ08-1609) have been systematically clarified [8,9,10], but the systematic biological basis of the excellent comprehensive performance of YT93-159 remains unclear, restricting its further germplasm innovation and targeted molecular breeding application.

In this review, we synthesize the breeding process, genetic characteristics, morphological phenotypes, cultivation adaptation characteristics, agronomic advantages, functional quality, and proposed molecular regulatory mechanisms of YT93-159 from the published literature and variety-registration records. We also compare YT93-159 with mainstream improved varieties across key agronomic and quality traits, and discuss its breeding value and industrial application prospects as a parental form. The review aims to provide theoretical support for the popularization and standardized cultivation of YT93-159, and to supply excellent germplasm resources and candidate gene targets for the multi-trait collaborative improvement of sugarcane varieties, and to offer a case study of the achievements and remaining challenges in South China sugarcane breeding. The evidence base and data provenance are described in Section 2.

Unlike prior variety descriptions that report agronomic performance as isolated records, this review links the field phenotype of YT93-159 to its underlying transcriptomic changes in carbon fixation, sucrose metabolism, phenylpropanoid biosynthesis, hormone signaling, and plant–pathogen interaction pathways. The transcriptomic findings are further translated into concrete breeding targets, including candidate genes for functional validation, molecular-marker development, and future wide hybridization.

## 2. Search Strategy and Data Sources

Because this manuscript is written as a narrative review with case-study elements, this section describes the literature search and the provenance of the evidence integrated below, so that previously published results, newly compiled analyses, and re-analyses of public datasets can be clearly separated.

The literature search was conducted in PubMed (https://pubmed.ncbi.nlm.nih.gov (accessed on 26 October 2026)), Web of Science (https://www.webofscience.com (accessed on 26 October 2026)), Scopus (https://www.scopus.com (accessed on 26 October 2026)), CNKI (https://www.cnki.net (accessed on 26 October 2026)), and Google Scholar (https://scholar.google.com (accessed on 26 October 2026)), using combinations of the following keywords: “sugarcane”, “Saccharum”, “YT93-159”, “Yuetang 93-159”, “sugar”, “sucrose”, “transcriptome”, “disease resistance”, “drought tolerance”, and “breeding”. The search covered studies published from 1993 to 2026 in both English and Chinese. Original research articles, systematic data reports, and variety-registration documents were prioritized; news items and non-peer-reviewed web content were excluded unless they served as the sole source for official statistical or registration information (as indicated below).

The data compiled in this review fall into four categories: first, peer-reviewed agronomic and molecular studies, including the transcriptomic and metabolomic analyses of sugarcane traits [6,7,11,12] and the systematic descriptions of mainstream varieties GT42, GT44, LC05-136, and YZ08-1609 [8,9,10]; second, variety-registration archives and regional-trial records of YT93-159, including its approval by the Guangdong Crop Variety Approval Committee (2002) and by the Ministry of Agriculture and Rural Affairs of China (2020), the multi-year field trial records (1996–1999), and the official variety description (unpublished data); third, statistical data from the National Information Center for Sugarcane Industry on national promotion acreage (Table 1); and fourth, previously published studies on cane juice quality [13], antioxidant activity [14], combining ability [15], and transcriptomic regulation of stress responses [11,16,17]. The raw RNA-seq data of sugarcane infected by smut (*Sporisorium scitamineum*) were deposited in the Genome Sequence Archive of the China National Center for Bioinformation (PRJCA011580) as reported in [16]; the baseline YT93-159-XTT22 comparison and the brown-rust time-course expression data were compiled from the authors’ earlier transcriptomic analyses [17], and the complete experimental procedures and differential-expression statistics of these datasets are reported in the corresponding original studies.

The cane-juice quality and antioxidant data cited in Section 6 were compiled from two comparative studies across seven and fourteen sugarcane varieties, respectively [13,14]. Li et al. [13] measured soluble solids, soluble sugars, titratable acids, soluble protein, polyphenols, and flavonoids, and ranked varieties by principal component and cluster analysis. Yang et al. [14] measured total polyphenols, total flavonoids, DPPH and ABTS radical-scavenging rates, total reducing power, FRAP, protein, reducing sugar, and sucrose content. In both studies, values are means ± SD from triplicate measurements, and varietal differences were tested by one-way ANOVA at *p* < 0.05. Detailed assay protocols are described in the original publications [13,14].

Where the original publications did not provide site- and year-level trial details or full differential-expression statistics, these limitations are noted explicitly in the relevant sections, and the corresponding conclusions are presented as reported findings or as hypotheses requiring further validation rather than as independently re-verified results.

## 3. Breeding Logic and Genetic Characteristics

### 3.1. Breeding Process

YT93-159 was independently bred by the Hainan Sugarcane Breeding Station of the former Guangzhou Sugarcane Industry Research Institute (renamed the Institute of Nanfan & Seed Industry, Guangdong Academy of Sciences in 2021), aiming to develop an early-maturing, high-yield, high-quality sugarcane variety with strong stress resistance to adapt to the climatic and cultivation conditions of southern China. The variety adopts the classic conventional hybrid breeding system with complementary parental trait design.

In 1992, artificial hybridization was carried out with Yuenong 73-204 (YN73-204) as the female parent and CP72-1210 as the male parent. The female parent YN73-204, bred by Guangdong Academy of Agricultural Sciences, has the typical advantages of a thick stem, high yield and strong lodging resistance, which can provide excellent yield and a plant-type genetic basis for hybrid progeny [18]. The male parent CP72-1210, selected and bred by the USDA Canal Point Sugarcane Breeding Program in Florida, USA, is famous for its early maturity, high sugar content and strong ratooning ability, which can effectively improve the sugar quality and early maturity of progeny [19]. The two parents have highly complementary agronomic traits, which lays a good genetic foundation for the formation of excellent comprehensive traits in YT93-159.

Hybrid seeds obtained from the hybridization were sown in the Zhanjiang Sugarcane Research Center of Guangdong Academy of Sciences in March 1993, and field transplanting of seedlings was completed in late May of the same year. In November 1993, single plant selection was carried out based on key agronomic traits including stem thickness, tillering performance and early sugar accumulation. From 1994 to 1999, the selected excellent single clone underwent five-stage systematic screening, including via a selection nursery, identification nursery, yield comparison trial, demonstration trial and production trial. From 1996 to 1999, multi-location regional adaptability trials were carried out in Guangdong, Guangxi and Hainan provinces, covering new planting and ratoon cane cropping modes, with the local mainstream commercial variety XTT10 as the control. The evaluation indices covered cane yield, sugar content, disease resistance and cultivation stability.

**Figure 1:**
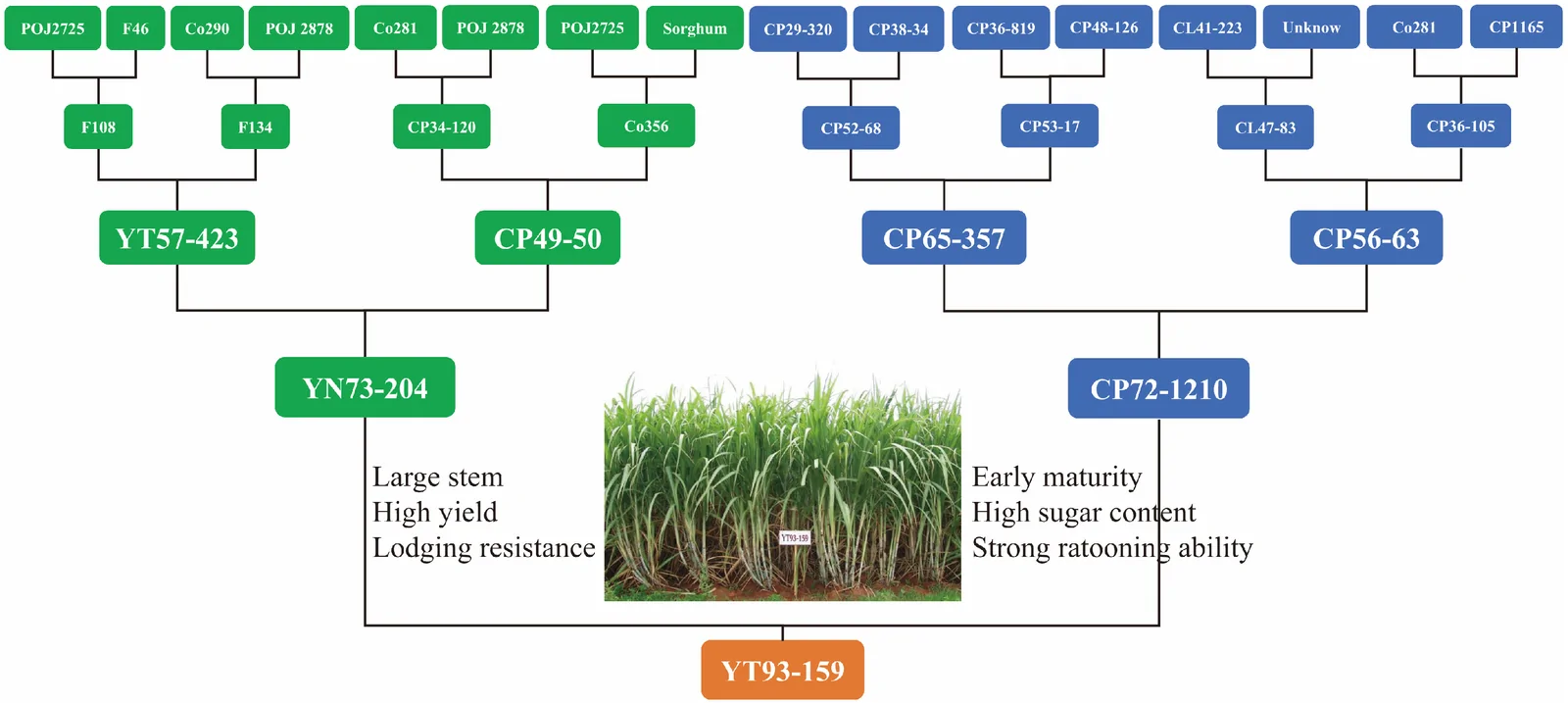
Pedigree and elite traits of sugarcane variety YT93-159.

After years of systematic screening and multi-point verification, YT93-159 was officially approved and released by the Guangdong Crop Variety Approval Committee in March 2002, and passed the national variety approval by the Ministry of Agriculture and Rural Affairs of China in June 2020, realizing the standardized popularization and application nationwide (Figure 1). Unless otherwise indicated, the breeding history, morphological description, and official release data in this section are drawn from the Guangdong provincial variety-registration record for YT93-159 (unpublished official document).

### 3.2. Morphological Traits

**Figure 2:**
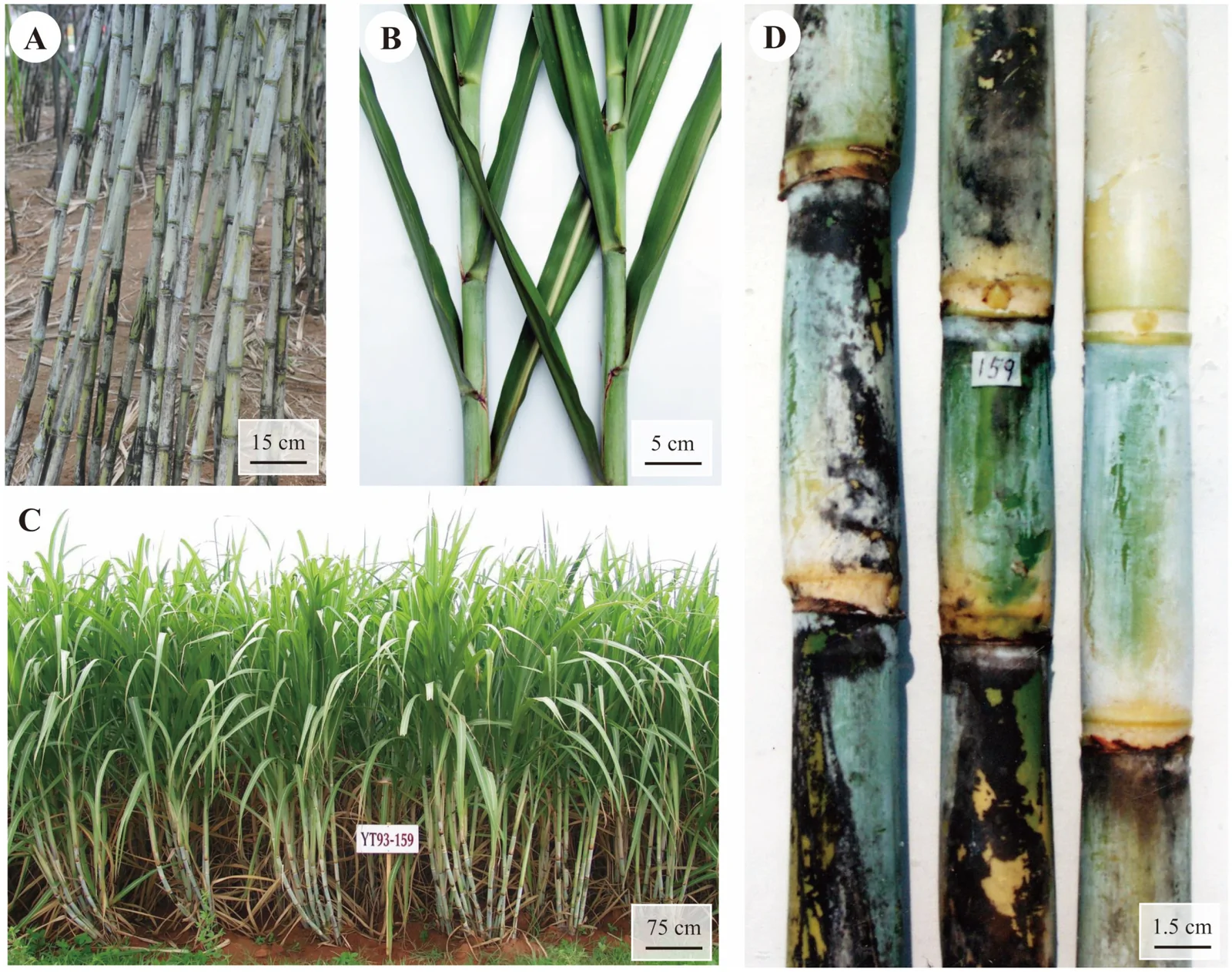
Genetic characteristics of sugarcane variety YT93-159. (**A**) plant type, (**B**) leaf, (**C**) maturity stage, (**D**) stem. Scale bars: 15 cm (**A**), 5 cm (**B**), 75 cm (**C**), 1.5 cm (**D**).

YT93-159 has stable and excellent morphological plant type characteristics, which are suitable for large-scale mechanized cultivation and field management (Figure 2). The variety presents medium-to-large stems with a stem diameter of 2.5–3.0 cm. The internodes are cylindrical and solid with slightly bulging basal parts, which endows the variety with good mechanical properties and lodging resistance. The stem color is yellowish-green in shaded environments and turns uniform greenish-yellow under natural sunlight, with neat internode arrangement and consistent plant growth.

The buds are oval, with the base closely attached to the leaf scar and the bud tip not exceeding the growth zone, showing strong germination potential. The leaves are light green; new leaves grow upright to improve light interception efficiency, while old leaves arch moderately to avoid excessive shading. The leaf sheath is greenish-yellow with moderate density of 57th hair groups. The inner leaf auricle is lanceolate and the outer one is triangular, with typical and stable variety morphological markers, which is convenient for field variety identification and seedling purification.

## 4. Standard Cultivation Practices

To maximize the yield and quality potential of YT93-159, standardized and targeted cultivation management measures are formulated based on its growth and development characteristics and ecological adaptability. Sowing time is suitable for winter (December to January of the subsequent year) and early-spring (February to March), which can make full use of soil temperature and moisture conditions to promote early germination and seedling growth. To adapt to mechanized sowing and field management, the optimal row spacing is set to 100–120 cm, with a sowing dosage of 84,000 double-internode buds per hectare. The reasonable population density is controlled at about 75,000 effective stems per hectare to balance individual growth and group yield formation. The choice of these sowing windows corresponds to the temperature requirements for sugarcane bud germination in southern China. Observations from the Guangxi Sugarcane Research Institute indicate that bud emergence is severely restricted below 13 °C, proceeds slowly below 20 °C, and is optimal between 25 and 32 °C [20]. Winter sowing (December–January) in Guangdong and Guangxi falls near the low-temperature end of this range, allowing slow root establishment rather than rapid shoot emergence; early-spring sowing (February–March) coincides with soil temperatures rising through 20–25 °C, where emergence rates improve substantially. Delaying sowing beyond March exposes setts to higher temperatures and erratic rainfall, which reduces bud survival and increases disease pressure on cut stems [20]. Cultivation parameters in this section are derived from the official variety cultivation technical regulations and regional-trial agronomic records.

Plastic film mulching is recommended in southern China, especially in high-altitude and low-temperature areas, and can effectively preserve soil temperature and moisture, accelerate seedling emergence, and improve seedling uniformity. In terms of fertilizer management, balanced application of nitrogen, phosphorus and potassium compound fertilizers is required, and excessive or single application of nitrogen fertilizer should be avoided to prevent excessive vegetative growth, delayed maturity and reduced sugar accumulation [21].

In pest and disease control, field monitoring of sugarcane smut and mosaic disease should be strengthened, and diseased plants should be removed in a timely manner to prevent pathogen spread and field infection. Stem borers and aphids, the main pests of sugarcane, are controlled by biological agents or low-toxicity and low-residue pesticides to ensure the green production of sugarcane. Notably, YT93-159 is sensitive to herbicide phytotoxicity; herbicide spraying should avoid splashing on leaf surfaces to prevent leaf damage and photosynthetic efficiency reduction, so as to ensure stable growth and yield formation.

## 5. Agronomic and Physiological Advantages

### 5.1. High Yield and Early High Sugar Accumulation

According to the Guangdong regional-trial and production-trial records of YT93-159, which are summarized in the official variety description (unpublished data), the regional and production trials conducted from 1996 to 1999, each comprising one new-planting and one ratoon crop, showed prominent yield advantages when averaged across these trials. The average cane yield of YT93-159 reached 102.42 t/ha, which was 13.99% higher than the control variety XTT10 (89.85 t/ha). Among them, the average yield of new-planted cane was 101.39 t/ha, 14.10% higher than the control; the average yield of ratoon cane was 103.46 t/ha, an increase of 13.89% compared with the control. The high yield performance of YT93-159 is mainly attributed to its strong tillering ability (75,000–90,000 effective stems per hectare) and rapid canopy closure. Rapid canopy formation can effectively inhibit weed growth, reduce nutrient competition, and improve canopy light interception and utilization efficiency, thereby promoting biomass accumulation and high yield formation [22]. For clarity, these trials comprised two independent two-year cycles (1996–1997 and 1998–1999) conducted at multiple locations across Guangdong, with both a plant-cane and a first-ratoon crop per cycle. The published variety record reports only the overall means averaged across cycles and locations; site-by-year tables, per-replication data, and formal ANOVA statistics are not included, and the means above should therefore be read as officially reported trial averages rather than as independently re-analyzed contrasts.

YT93-159 has the typical characteristics of early maturity and high sugar accumulation, which can effectively advance the sugarcane harvesting and processing period and extend the industrial production cycle. In the regional and production trials, its sugar content was 15.55% in November, 16.38% in December, and 17.06% in January, consistently above the control XTT10 (14.12%, 14.74%, 15.10%) over the same period. The mean sugar content from November to January was 16.33%, 1.68 percentage points above the control (14.65%). On a per-hectare basis, sugar yield reached 16.73 t/ha, 27.07% higher than the control (13.17 t/ha).

Further classification statistics showed that new-planted cane had better sugar accumulation performance: the sugar content was 15.42% in November, 16.11% in December and 16.85% in January, with an average of 16.13% in three months, 1.67 percentage points higher than the control. The ratoon cane also showed stable high-sugar characteristics, with an average sugar content of 16.53% from November to January, 1.68 percentage points higher than XTT10. This early and efficient sugar accumulation overcomes the low-sugar limitation of conventional early-maturing varieties and confers substantial industrial application value. Because the original trial records do not report site- and year-level statistical models or uncertainty estimates for these comparisons, the differences above should be read as reported trial means rather than as independently verified statistical contrasts.

### 5.2. Excellent Multi-Stress Resistance

YT93-159 has excellent biotic and abiotic stress resistance, with stable field performance and low yield loss under adverse environments. In terms of disease resistance, artificial inoculation identification and long-term field observation reported in the variety-registration documents confirmed that YT93-159 has high resistance to sugarcane smut (resistance grade 1) and mosaic disease (resistance grade 2), and no obvious infection of yellow spot disease, brown streak disease and rust disease was observed in the field, indicating broad-spectrum disease resistance under the tested conditions. Resistance grades in this section are from the official variety-registration field observation and artificial inoculation identification records.

In terms of abiotic stress resistance, YT93-159 has strong cold tolerance and drought tolerance. Under drought and low-temperature stress, the variety can maintain stable physiological metabolism, with reportedly insignificant yield reduction [23]. In addition, YT93-159 has solid stems and low plant gravity center, forming a good mechanical lodging resistance structure. Under typhoon and strong wind conditions, the wind breakage rate is less than 5%, which is reported to be superior to most conventional varieties, and is suitable for planting in coastal typhoon-prone areas of southern China [24]. As with the yield data, the formal experimental design, replication, and statistical analyses underlying these resistance assessments are not fully detailed in the published record and therefore cannot be independently re-evaluated here.

### 5.3. Wide Regional Adaptability and Large-Scale Popularization Performance

YT93-159 has wide ecological adaptability, suitable for popularization and planting in Guangdong, Guangxi, Hainan, Yunnan and other major sugarcane-producing areas in southern China. It can adapt to both irrigated and rain-fed cultivation conditions, and performs best in medium and high-fertility farmland soil. It can be sown in winter and spring, and plastic film mulching can further optimize soil temperature and moisture conditions, promote seedling growth, and improve population stability. Promotion-area statistics are compiled from the National Information Center for Sugarcane Industry (government compilation).

According to the statistical data of the National Information Center for Sugarcane Industry, from 2021 to 2025, the cumulative national promotion area of YT93-159 was 256,234 hectares, accounting for 4.91% of China’s total sugarcane planting area, ranking among the top five mainstream sugarcane varieties in China. The annual planting area remained stable at more than 37,000 hectares, showing good market recognition and promotion potential (Table 1).

## 6. Sugarcane Juice Quality and Antioxidant Activity

### 6.1. Nutritional Quality Indicators of Cane Juice

According to the comparative juice-quality study reported in [13], YT93-159 has excellent cane juice nutritional quality, with advantages in soluble solids, polyphenols, flavonoids and other functional quality indicators compared with the other tested sugarcane varieties. The soluble solids content of YT93-159 cane juice reaches 21.00%, which is significantly higher than that of the tested varieties such as Dianzhe 01-104 and purple cane. Its polyphenol content is 1.21 g/L, flavonoid content is 0.58%, and protein content is 0.27%, all of which are at a high level among tested varieties. Principal component analysis of quality indicators in the same study showed that the comprehensive quality score of YT93-159 was 47.72, ranking first among the seven tested sugarcane varieties, indicating that YT93-159 has prominent comprehensive advantages in cane juice nutritional quality and functional components (Table 2).

### 6.2. Antioxidant Activity and Functional Application Potential

YT93-159 cane juice has rich antioxidant active substances and strong free radical scavenging capacity, with excellent functional food development value [25]. According to the comparative analysis reported in [14], the total polyphenol content of YT93-159 cane juice is 878.22 mg/kg, total flavonoid content is 830.45 mg/kg, DPPH radical scavenging rate is 83.02%, ABTS radical scavenging rate is 71.31%, and total reducing power is 1.16, showing excellent antioxidant comprehensive performance (Table 3). The brown sugar processed from YT93-159 ranks second in the comprehensive quality evaluation of 14 sugarcane varieties based on the entropy-weighted TOPSIS method, with outstanding nutritional and antioxidant quality [14].

Further studies showed that the organic acid extract of YT93-159 has strong antioxidant activity, with excellent performance in DPPH, FRAP and PFRAP detection methods, second only to the high-quality functional variety XTT16. Transcriptome and metabolome combined analysis confirmed that YT93-159 contains a large number of candidate genes related to organic acid and specific amino acid biosynthesis, and the gene expression pattern is highly consistent with metabolite accumulation characteristics [26]. The rich accumulation of organic acids, polyphenols and flavonoids endows YT93-159 with excellent antioxidant function, making it a high-quality raw material for natural antioxidant and functional sugar food development.

The high antioxidant capacity of YT93-159 cane juice is consistent with the broader physiological literature on oxidative-stress tolerance in sugarcane. In a drought-tolerant sugarcane variety, water deficit reduces leaf relative water content and elevates malondialdehyde, while coordinated changes in SOD, POD, and CAT activities constitute the primary enzymatic defense against drought-induced ROS accumulation [23]. Under low-temperature stress, cold-acclimated plants rely on the same SOD–CAT–POD axis together with proline accumulation for osmotic adjustment and membrane stabilization [27]. These mechanisms align with the biochemical data in Table 2 and Table 3: the high basal polyphenol and flavonoid pool in YT93-159 (878.22 mg/kg and 830.45 mg/kg) provides direct free-radical scavenging while also potentially inducing the inducible enzymatic ROS-scavenging systems described above, coupling constitutive non-enzymatic antioxidants with inducible enzymatic defense.

## 7. Molecular Mechanisms Underlying Elite Agronomic Traits

The molecular information summarized in this section was compiled from the authors’ previously published transcriptomic analyses [17] and the associated public RNA-seq dataset (PRJCA011580) [16], which compared YT93-159 with the conventional variety XTT22 under baseline, brown-rust, and smut-challenge conditions. The experimental design, tissue and developmental-stage information, sequencing procedures, normalization method, and differential-expression criteria of these datasets are reported in the corresponding original publications, and the complete differential-expression statistics (including log2 fold changes and adjusted *p*-values) should be consulted in those sources. Because the transcriptomic evidence is correlative and several genes within a given pathway showed opposing expression changes, the pathway-level interpretations below are presented as candidate hypotheses that require functional validation, rather than as established causal mechanisms.

### 7.1. Transcriptomic Characteristics of Core Regulatory Pathways

**Figure 3:**
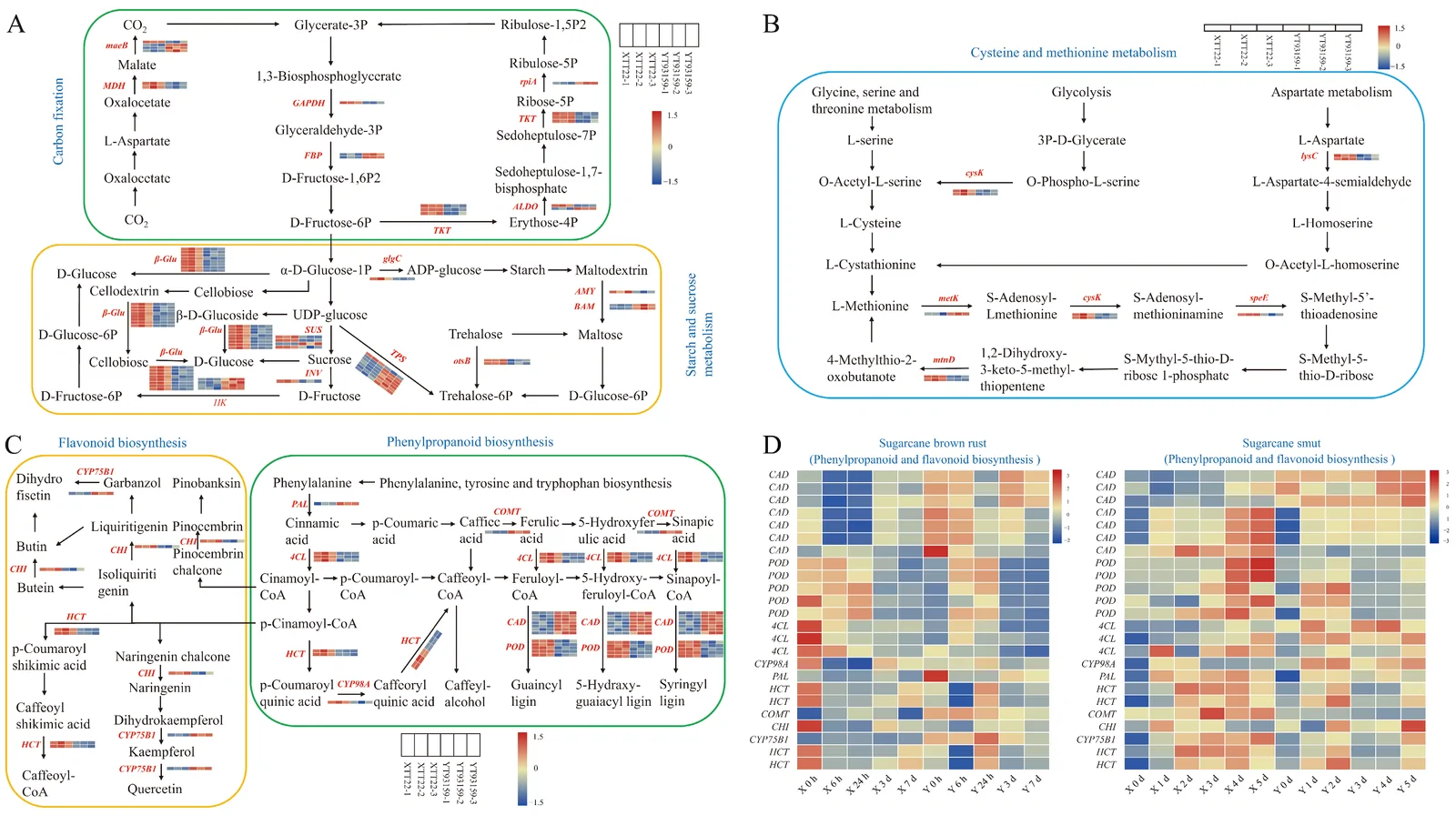
Transcriptomic pathway enrichment and differential gene expression profiles of YT93-159. Key pathways including (**A**) carbon fixation and starch/sucrose metabolism, (**B**) cysteine and methionine metabolism, (**C**) phenylpropanoid and flavonoid biosynthesis, with color scales representing gene expression levels compared with XTT22. The heat maps represent the expression of DEGs in YT93-159 compared with the control variety. Red and blue indicate up-regulated and down-regulated genes, respectively. (**D**) The expression pattern of pivotal genes within the phenylpropanoid and flavonoid biosynthesis pathway. X-(0 h, 6 h, 24 h, 3 d, 7 d) and Y-(0 h, 6 h, 24 h, 3 d, 7 d) represent XTT22 (susceptible) and YT93-159 (tolerant) suffered from sugarcane brown rust for 0 h, 6 h, 24 h, 3 d, 7 d, respectively. X-(0 d, 1 d, 2 d, 3 d, 4 d, 5 d) and Y-(0 d, 1 d, 2 d, 3 d, 4 d, 5 d) represent 1 to 5 d for XTT22 and YT93-159 post smut (*Sporisorium scitamineum*) inoculation, respectively. maeB, malate dehydrogenase (oxaloacetate-decarboxylating) (NADP+); MDH, malate dehydrogenase; GAPDH, glyceraldehyde 3-phosphate dehydrogenase (phosphorylating); FBP, fructose-1,6-bisphosphatase I; TKT, transketolase; rpiA, ribose 5-phosphate isomerase A; ALDO, fructose-bisphosphate aldolase; β-Glu, beta-glucosidase; HK, hexokinase; SUS, sucrose synthase; INV, beta-fructofuranosidase; glgC, glucose-1-phosphate adenylyltransferase; TPS, trehalose 6-phosphate synthase/phosphatase; otsB, trehalose 6-phosphate phosphatase; AMY, alpha-amylase; BAM, beta-amylase; cysK, cysteine synthase A; metK, S-adenosylmethionine synthetase; mtnD, 1,2-dihydroxy-3-keto-5-methylthiopentene dioxygenase; lysC, bifunctional aspartokinase/homoserine dehydrogenase 1; speE, spermidine synthase; CYP75B1, flavonoid 3′-monooxygenase; CHI, chalcone isomerase; HCT, shikimate O-hydroxycinnamoyl transferase; PAL, phenylalanine ammonia-lyase; 4CL, 4-coumarate-CoA ligase; CYP98A, coumaroylquinate (coumaroylshikimate) 3′-monooxygenase; COMT, caffeic acid 3-O-methyltransferase; CAD, cinnamyl-alcohol dehydrogenase; POD, peroxidase. Arrows indicate the direction of enzymatic reactions or metabolic flux. Supplementary Materials File S1 lists the transcriptomic expression levels of the differentially expressed genes shown here.

In the compiled transcriptomic datasets, differentially expressed genes (DEGs) of YT93-159 relative to XTT22 were enriched in carbon fixation, starch and sucrose metabolism, phenylpropanoid and flavonoid biosynthesis, amino acid metabolism, plant hormone signal transduction and plant–pathogen interaction pathways, forming a multi-layered regulatory landscape for yield formation, quality improvement and stress resistance [7,11,16,17]. Importantly, within each of these pathways, both up-regulated and down-regulated members were observed, and the net functional consequences of these opposing expression changes are not yet resolved; we therefore describe the expression patterns gene by gene and treat the pathway-level interpretations as hypotheses. In the carbon fixation pathway (Figure 3A), core functional genes such as malate dehydrogenase (*maeB*), fructose-1,6-bisphosphatase I (*FBP*), and ribose 5-phosphate isomerase A (*rpiA*) were up-regulated in YT93-159 compared with the control, while malate dehydrogenase (*MDH*), glyceraldehyde 3-phosphate dehydrogenase (*GAPDH*), and transketolase (*TKT*) were down-regulated, and fructose-bisphosphate aldolase (*ALDO*) showed complex differential expression patterns. The co-occurrence of up- and down-regulated members suggests that carbon-fixation capacity may be enhanced in a gene-specific manner, which could provide carbon skeletons for sucrose synthesis and secondary metabolite accumulation [12]; this possibility remains to be functionally tested.

In the starch and sucrose metabolism pathway (Figure 3A), hexokinase (*HK*), trehalose 6-phosphate synthase/phosphatase (*TPS*), and beta-amylase (*BAM*) genes were up-regulated, whereas key genes such as beta-glucosidase (*β-Glu*), sucrose synthase (*SUS*), beta-fructofuranosidase (*INV*), glucose-1-phosphate adenylyltransferase (*glgC*), trehalose 6-phosphate phosphatase (*otsB*), and alpha-amylase (*AMY*) were down-regulated. Because *SUS* and *INV*, which encode the major sucrose-metabolizing enzymes, showed lower expression in YT93-159, the induced expression of *HK*, *TPS* and *BAM* cannot by itself be interpreted as a uniform activation of sucrose accumulation. The opposing expression of these paralogs makes the net direction of early sucrose metabolism uncertain, and the proposed link to the early-maturing and high-sugar phenotype should be regarded as a hypothesis requiring experimental validation.

Amino acid metabolism, especially cysteine and methionine metabolism, was also differentially regulated in YT93-159 (Figure 3B). The up-regulation of the S-adenosylmethionine synthetase (*metK*) gene may promote the synthesis and accumulation of sulfur-containing amino acids, which could provide nutritional support for the synthesis of stress-resistant functional proteins and secondary metabolites, while the differential expression of cysteine synthase A (*cysK*), 1,2-dihydroxy-3-keto-5-methylthiopentene dioxygenase (*mtnD*), bifunctional aspartokinase/homoserine dehydrogenase 1 (*lysC*), and spermidine synthase (*speE*) may maintain the metabolic balance of amino acids and ensure stable plant growth [28].

The phenylpropanoid and flavonoid biosynthesis pathways showed mixed expression changes in YT93-159 (Figure 3C). Core genes including phenylalanine ammonia-lyase (*PAL*), caffeic acid 3-O-methyltransferase (*COMT*), cinnamyl-alcohol dehydrogenase (*CAD*), and flavonoid 3′-monooxygenase (*CYP75B1*) were up-regulated, while 4-coumarate-CoA ligase (*4CL*), shikimate O-hydroxycinnamoyl transferase (*HCT*), peroxidase (*POD*), coumaroylquinate (coumaroylshikimate) 3′-monooxygenase (*CYP98A*), and chalcone isomerase (*CHI*) were down-regulated. The up-regulation of *PAL*, *COMT* and *CAD* is consistent with a possible stimulation of the early phenylpropanoid and monolignol branches and with the high accumulation of polyphenols and flavonoids in YT93-159 cane juice [29]; however, the concurrent down-regulation of *4CL*, *HCT* and *POD* complicates a simple activation model for this pathway, and the functional significance of these opposing changes for antioxidant capacity, cell-wall reinforcement, and resistance to fungal diseases such as brown rust and smut (Figure 3D) requires direct experimental assessment.

### 7.2. Plant Hormone Signal Transduction and Growth-Stress Balance Mechanism

**Figure 4:**
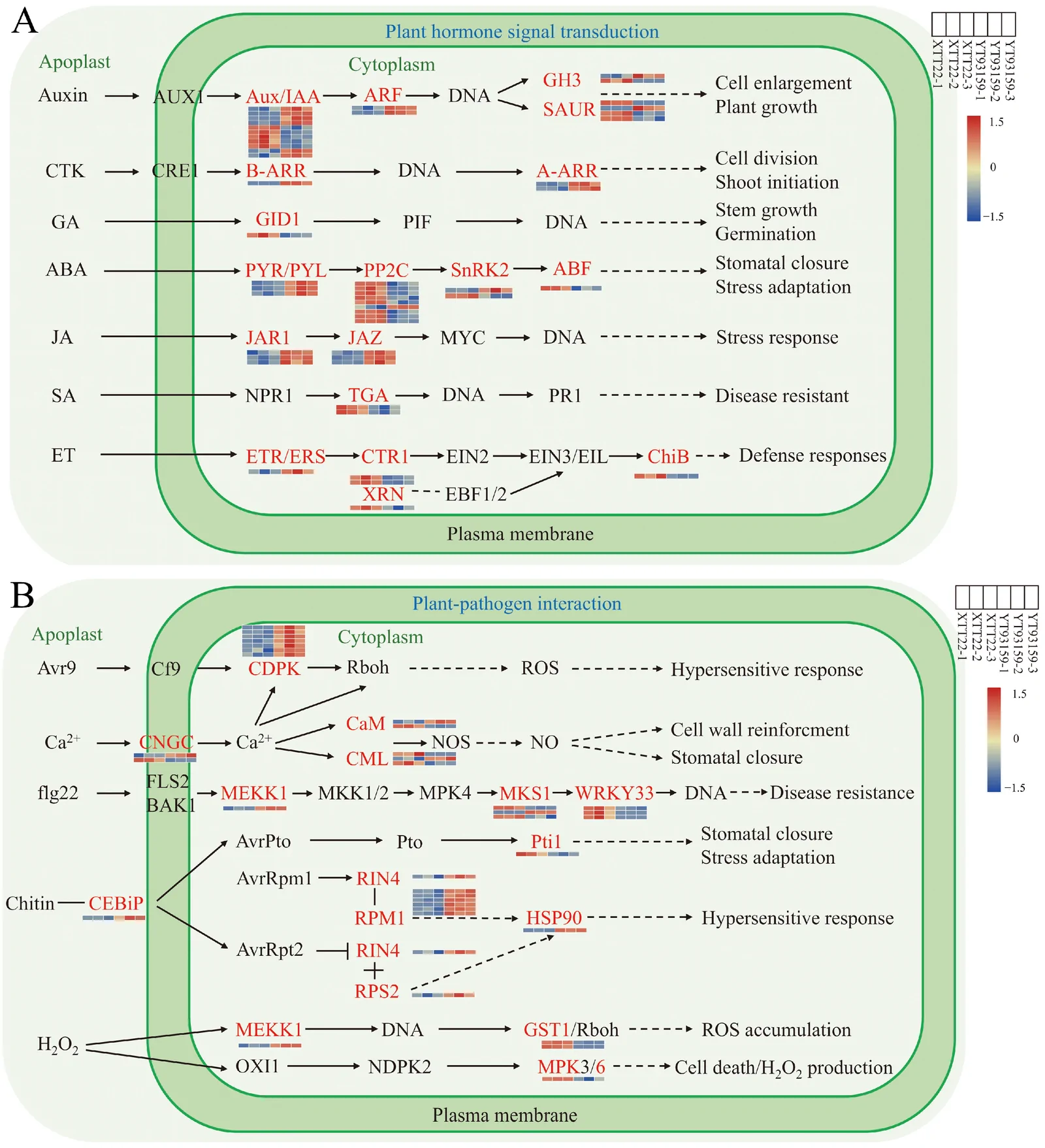
Transcriptomic pathway enrichment and differential gene expression profiles of YT93-159. Key pathways including (**A**) plant hormone signal transduction and (**B**) plant–pathogen interaction, with color scales representing gene expression levels compared with XTT22. The heat maps represent the expression of DEGs in YT93-159 compared with the control variety. Red and blue indicate up-regulated and down-regulated genes, respectively. AUX/IAA, auxin; ARF, auxin response factor; GH3, gretchen hagen 3; SAUR, small auxin up-regulated RNA; A/B-ARR, type-A/B Arabidopsis response regulator; GID1, GIBBERELLIN INSENSITIVE DWARF 1; PYR/PYL, abscisic acid receptor PYR/PYL family; PP2C, protein phosphatase 2C; SnRK2, serine/threonine-protein kinase 2; ABF, ABA responsive element binding factor; JAR1, jasmonic acid-amino synthetase 1; JAZ, jasmonate ZIM domain-containing protein; TGA, transcription factor TGA; ETR/ERS, ethylene receptor; CTR1, serine/threonine-protein kinase CTR1; ChiB, chitinase B; XRN, 5′-3′ exoribonuclease 4; CDPK, calcium-dependent protein kinase; CNGC, cyclic nucleotide gated channel, other eukaryote; CaM, calmodulin; CML, calcium-binding protein CML; MEKK1, mitogen-activated protein kinase kinase kinase 1; MKS1, MAP kinase substrate 1; WRKY33, WRKY transcription factor 33; CEBiP, chitin elicitor-binding protein; PTI1, pto-interacting protein 1; RIN4, RPM1-interacting protein 4; RPM1, disease resistance protein RPM1; RPS2, disease resistance protein RPS2; HSP90, heat shock protein 90 kDa; GST1, glutathione S-transferase 1; MPK6, mitogen-activated protein kinase 6. Arrows indicate the direction of signal transduction or regulatory relationships.

Plant hormone signal transduction is proposed to be a core regulatory module for YT93-159 to coordinate growth development and stress adaptation (Figure 4A). In auxin, cytokinin (CTK) and gibberellin (GA) signaling pathways, multiple DEGs such as auxin (*AUX/IAA*), auxin response factor (*ARF*), gretchen hagen 3 (*GH3*), small auxin up-regulated RNA (*SAUR*), type-A/B Arabidopsis response regulator (*A/B-ARR*), and GIBBERELLIN INSENSITIVE DWARF 1 (*GID1*) were up-regulated in the compiled datasets. The activation of auxin signaling may promote cell elongation and leaf expansion; CTK signaling may accelerate tiller bud differentiation, which could contribute to the strong tillering ability of YT93-159; GA signaling may promote rapid canopy closure, improve photosynthetic light-use efficiency, and suppress weed growth. Together, these observations provide a transcriptional for high-yield formation, although causal relationships remain to be established.

In abscisic acid (ABA), jasmonic acid (JA) and salicylic acid (SA) signaling pathways, a series of stress-responsive genes including PYR/PYL family (*PYR/PYL*), protein phosphatase 2C (*PP2C*), serine/threonine-protein kinase 2 (*SnRK2*), ABA responsive element binding factor (*ABF*), jasmonic acid-amino synthetase 1 (*JAR1*), jasmonate ZIM domain-containing protein (*JAZ*), transcription factor TGA (*TGA*), ethylene receptor (*ETR/ERS*), serine/threonine-protein kinase CTR1 (*CTR1*), chitinase B (*ChiB*), and 5′-3′ exoribonuclease 4 (*XRN*) were differentially expressed. Based on the well-characterized functions of these genes in other species [30,31], the ABA signaling module composed of PYR/PYL-PP2C-SnRK2-ABF may induce stomatal closure, reduce transpirational water loss, and activate downstream stress protective proteins, which could contribute to the drought and cold tolerance of YT93-159. JA and SA signaling pathways are likely activated under pathogen stress, triggering downstream immune defense responses and forming a hormone-mediated disease resistance network [27]. These interpretations are consistent with, but not directly proven by, the transcriptomic data.

Notably, YT93-159 is proposed to have a sophisticated hormone signal crosstalk mechanism. Under suitable growth conditions, GA and CTK signals may dominate growth regulation to ensure high biomass accumulation; under adverse stress conditions, ABA signal may moderately inhibit vegetative growth, and redistribute carbon assimilates to sucrose synthesis and defense metabolite accumulation, achieving a dynamic balance between high yield and stable stress resistance. This crosstalk model, although plausible, has not yet been experimentally confirmed in YT93-159.

### 7.3. Plant–Pathogen Interaction and Disease Resistance Mechanism

The high resistance of YT93-159 to smut, mosaic disease and brown rust has been proposed to be associated with the dual-layer immune system of PAMP-triggered immunity (PTI) and effector-triggered immunity (ETI) in the plant–pathogen interaction pathway (Figure 4B). After pathogen infection, the pattern recognition receptor gene chitin elicitor binding protein (*CEBiP*) was sharply up-regulated in the compiled dataset, which may recognize the chitin component of fungal cell walls and recruit the co-receptor BAK1 to initiate early immune signal transmission [31,32,33].

Subsequently, cyclic nucleotide-gated channel genes (*CNGCs*) and calcium-dependent protein kinase (*CDPK*) genes were induced, which may trigger intracellular Ca^2+^ burst as a secondary messenger to activate the downstream MEKK1-MKK1/2-MPK4 cascade pathway [32,34]. This pathway may negatively regulate the expression of *WRKY33*, avoiding excessive immune response and self-inflicted cellular damage, and further activate the expression of defense proteins such as pathogenesis-related protein genes (*PR*) and chitinase (*ChiB*), which may degrade fungal cell walls and inhibit pathogen colonization and expansion [16,35].

In the ETI immune pathway, the resistance protein genes *RPM1* and *RPS2* were up-regulated, which may trigger localized hypersensitive cell death to block pathogen spread. Meanwhile, ROS-scavenging genes such as superoxide dismutase (*SOD*), catalase (*CAT*), and ascorbate peroxidase (*APX*) were differentially expressed (Figure S1). Notably, several *CAT* and *APX* copies showed lower expression in YT93-159, so the overall direction of ROS-scavenging regulation is not uniform across paralogs; whether the net effect maintains intracellular ROS balance and confines ROS bursts to pathogen infection sites requires further analysis. These immune-related expression changes are regarded as candidate molecular correlates of the observed disease resistance, pending functional validation.

The transcriptomic evidence summarized in this section is derived specifically from YT93-159 (brown-rust tolerant, smut resistant) compared with the conventional control variety XTT22 (susceptible), dataset PRJCA011580 [16,17]; these findings should therefore be read as genotype-associated expression variation rather than as universally applicable resistance mechanisms. The PTI module (CEBiP–BAK1 chitin recognition, CNGC/CDPK Ca^2+^ signaling, MEKK1–MKK1/2–MPK4 cascade, *WRKY33*) and the ETI module (*RPM1/RPS2*, hypersensitive response) are broadly conserved across Arabidopsis, rice, and other sugarcane genotypes [31,32,33,34,35]. What differs in YT93-159 is the timing and amplitude of induction: in the brown-rust time-course (Figure 3D), *CEBiP*, *CNGC*, and *CDPK* transcripts accumulate earlier and more strongly in YT93-159 than in XTT22 across the 0 h–7 d infection window. Whether this stronger induction is shared with other resistant varieties remains to be tested by direct multi-genotype comparison.

For breeding applications, the most actionable genes are those that show consistent, directionally coherent expression changes and directly map to traits that breeders select. The up-regulated *HK*, *TPS*, and *BAM* genes are the strongest candidates for marker-assisted selection of early sugar accumulation, because their induction coincides with the early-maturing phenotype and they catalyze steps immediately upstream of sucrose turnover. Conversely, the down-regulation of *SUS* and *INV* complicates a straightforward sucrose-accumulation model; these two genes should be treated as candidates for further functional validation rather than as usable markers at this stage. In the phenylpropanoid pathway, the up-regulated *PAL*, *COMT*, and *CAD* genes are plausible targets for metabolic engineering aimed at raising polyphenol and flavonoid content, but the concurrent down-regulation of *4CL* and *HCT* means that overexpressing any single enzyme may not redirect flux as expected. In the immune module, *CEBiP*, *RPM1*, and *RPS2* are candidate markers for disease-resistance screening, given their stronger and earlier induction in YT93-159 than in XTT22 during pathogen challenge. The complete log2 fold-changes and adjusted *p*-values for all these genes are provided in Supplementary File S1 and in the original datasets [16,17].

### 7.4. Synergistic Formation Mechanism of Early Maturity, High Quality and Stress Resistance

The excellent comprehensive traits of YT93-159 may be formed by the synergistic regulation of multiple pathways, but the evidence for each link remains indirect. For early maturity and high sugar accumulation, auxin and GA signaling may enhance photosynthetic carbon fixation capacity and increase carbon source supply. WRKY and MYB transcription factors activated by immune signaling may bind to the promoters of sucrose metabolism genes *SUS* and *INV* [36], through epigenetic histone acetylation [37], to up-regulate the transcription of sucrose synthesis-related genes in the early growth stage, enabling early and efficient sucrose accumulation [38]. However, because *SUS* and *INV* were down-regulated in the compiled transcriptomic datasets, the regulatory model proposed here needs to be reconciled with these observations through targeted experiments.

The high accumulation of polyphenols and flavonoids may be derived from the synergistic activation of plant–pathogen interaction and phenylpropanoid biosynthesis pathways. Immune-activated transcription factors may directly promote the expression of core genes such as *PAL* and *COMT*, driving the synthesis of lignin, flavonoids and polyphenols [29,39]. Lignin deposition may thicken cell walls to form a physical defense barrier against pathogens [40], while flavonoids and polyphenols may have direct antifungal and antioxidant effects, potentially combining disease resistance improvement with nutritional quality optimization (high polyphenol and flavonoid content as shown in Table 2 and Table 3).

Under drought or cold stress, ABA signaling genes (*PYR/PYL*, *PP2C*) were up-regulated in the compiled datasets, which may regulate stomatal closure and water retention [41]. The SnRK2 kinase, activated upon ABA binding to PYR/PYL receptors, may phosphorylate downstream transcription factors such as *ABF4*, which may induce the expression of drought-responsive genes including *RD29A* and *COR47* [42]. Simultaneously, high polyphenol/flavonoid levels (878.22 mg/kg and 830.45 mg/kg) may scavenge free radicals and protect cellular structures [14]. This synergy may maintain juice quality stability even under stress, ensuring uninterrupted sugar accumulation. As with the other molecular interpretations, these links are based on gene-expression correlations and the known functions of the corresponding genes in other plants, and they have not been directly validated in YT93-159.

In terms of stress-quality synergy, the phenylpropanoid metabolic pathway may act as a shared core module for biotic stress resistance, abiotic stress adaptation and quality formation. The high accumulation of secondary metabolites may enhance the multi-stress tolerance of YT93-159 and improve the functional nutritional value of cane juice, potentially realizing the collaborative optimization of stress resistance and quality traits.

### 7.5. Seed Germination and Growth Regulation Mechanism

YT93-159 has unique environmental response characteristics in seed germination and early growth, as reported in a transcriptomic study of ultrasonic treatment [43]. Ultrasonic treatment (20–40 kHz, 5 min) up-regulates GA synthesis genes (*GA20ox* and *GA3ox*) while down-regulating ABA biosynthesis genes (*NCED*), increasing the IAA/ABA ratio to promote germination [43]. The DELLA proteins, negative regulators of GA signaling, are degraded upon GA perception, releasing their inhibition on growth-promoting genes [44]. Concurrently, antioxidant enzyme genes (*SOD*, *POD*) and energy metabolism genes (ATP synthases) are activated, boosted by the up-regulation of the NAC transcription factor, which binds to their promoter regions [30]. Such transcriptional reprogramming may elevate soluble protein accumulation and mitigate oxidative damage during seedling establishment. This finely tuned regulatory program may guarantee rapid and uniform germination under favorable conditions and prevent precocious sprouting under suboptimal environments, thereby stabilizing field population structure.

## 8. Breeding Value of YT93-159

YT93-159 is an excellent core parent of Yuetang series sugarcane varieties, with prominent general combining ability and special combining ability, especially suitable for the genetic improvement of high-sugar and high-yield traits. Our previous study on 6 Yuetang parental materials and 55 hybrid combinations [15] showed that YT93-159 has the highest general combining ability (GCA) effect value for sugar content as a male parent, with a positive GCA effect (0.02), which can stably transmit high-sugar traits to hybrid progeny. Its economic breeding value (EBV) as a male parent reaches 269.72, indicating excellent breeding application potential.

In excellent hybrid combinations, Guitang 02-467 × YT93-159 showed positive special combining ability (SCA) effects for both sugar content (1.14) and cane yield (5.94), with an EBV of 311.99, ranking among the top three of all tested combinations. The XTT22 × YT93-159 combination also performed well, with positive sugar content SCA effect (0.98) and EBV of 250.04. These results verify that YT93-159 can significantly improve the comprehensive agronomic performance of hybrid progeny, and is an excellent parent for high-sugar sugarcane breeding (Table 4).

Compared with mainstream improved varieties in China such as GT42, GT44, LC05-136 and YZ08-1609 [8,9,10], YT93-159 has unique breeding advantages in early sugar accumulation and secondary metabolite accumulation. While the fifth-generation GT series varieties have outstanding mechanized adaptability and ratooning performance, YT93-159 fills the gap in early-maturity and high-quality traits among mainstream varieties. The unique sucrose metabolism and phenylpropanoid pathway regulation patterns of YT93-159 provide new gene resources and metabolic modules for multi-trait collaborative improvement in sugarcane breeding. A critical comparison of YT93-159 with these mainstream varieties across key traits is summarized in Table 5.

## 9. Challenges, Future Prospects and Breeding Strategy

### 9.1. Existing Limitations and Planting Challenges

Although YT93-159 has excellent comprehensive performance, it still has some limitations in production and application. Firstly, YT93-159 is sensitive to herbicide phytotoxicity, which is related to the low expression of herbicide-metabolizing cytochrome P450 family genes in vivo [46,47]. This trait requires more careful field weed management and limits its large-scale promotion in labor-shortage areas. Secondly, YT93-159 performs best in medium- and high-fertility soil, and its adaptability to low-fertility marginal land is insufficient, which restricts its planting range expansion. Thirdly, with climate change, new pathogenic races and extreme high-temperature and drought stresses continue to appear, which may break the existing resistance mechanism of YT93-159 and affect its stable yield and quality.

In addition, two evidence-related limitations should be acknowledged. First, the agronomic and resistance data cited above are largely derived from regional-trial records and variety-registration archives; because site- and year-level results, replication, and statistical models are not fully reported in the published sources, the corresponding comparisons should be treated as reported trial means. Second, the molecular mechanisms summarized in Section 7 are based on published differential-expression and pathway-enrichment analyses without complete log2 fold-change and adjusted *p*-value tables in this manuscript; the full statistics are available in the original studies and public datasets, and the pathway-level interpretations remain candidate hypotheses until functionally validated.

### 9.2. Future Perspectives and Breeding Strategy

The early maturity trait of YT93-159 can extend the sugarcane processing cycle by 3-4 weeks, increasing the annual sugar output of processing enterprises by about 12%, with significant industrial economic benefits. To address these deficiencies, targeted improvement and innovation can be made through molecular breeding and cultivation optimization.

From a breeding-strategy perspective, the candidate regulatory genes identified from the transcriptomic analyses provide a starting point for future genomic breeding. For herbicide tolerance, CRISPR gene editing technology could be used to modify P450 enzyme genes such as *CYP81A* to improve herbicide tolerance and reduce the labor requirement for field management [46,47]. For the phenylpropanoid and flavonoid traits, targeted metabolic engineering of the phenylpropanoid pathway has been proposed to further increase polyphenol and flavonoid content by 15–20% and simultaneously improve disease resistance and functional quality. To realize this goal, the candidate genes up-regulated in YT93-159 (e.g., *PAL*, *COMT* and *CAD*) and the down-regulated members (e.g., *4CL*, *HCT* and *POD*) should first be functionally characterized across tissues and developmental stages to establish which genes are suitable metabolic-engineering targets; given that *4CL* showed lower expression in the compiled transcriptomic datasets, its suitability as an overexpression target requires experimental assessment before it is adopted. Optimizing ultrasonic germination treatment parameters can standardize seedling emergence technology and adapt to large-scale mechanized production [43].

For disease resistance and yield, the transcriptome-derived candidate genes (e.g., immune-related transcription factors such as *WRKY33*, and resistance-associated genes) could be prioritized for functional validation and for the development of molecular markers. Developing molecular markers linked to sugar content, disease resistance and yield traits of YT93-159 can accelerate marker-assisted selective breeding (MAS), shorten the breeding cycle, and support the continued development of new elite varieties. In terms of functional application, the excellent antioxidant quality of YT93-159 lays a foundation for the development of functional sugar and nutraceutical products. In terms of germplasm innovation, interspecific hybridization with wild sugarcane resources such as *S. spontaneum* can introduce drought resistance and pest resistance alleles, improve nutrient utilization efficiency, and expand its adaptability to marginal land.

In conclusion, YT93-159 is an excellent early-maturing, high-yield and high-quality sugarcane variety integrating yield, quality and resistance. The combination of conventional breeding and molecular omics technology will further tap its germplasm potential, promote the sustainable development of the sugarcane industry in tropical and subtropical regions, and provide a model for the collaborative improvement of multiple agronomic traits in sugarcane.

## Figures and Tables

**Table 1:** Information on the trend in promotion acreage for YT93-159 in China.

Metric	2021	2022	2023	2024	2025	Total
Planting area (hectare)	71,987	46,567	44,653	55,220	37,807	256,234
Percentage of total area (%)	6.62	4.61	4.46	5.42	3.45	4.91
Ranking	4	4	4	5	5	

Note: The data were provided by the National Information Center for Sugarcane Industry.

**Table 2:** Sugarcane juice quality indicators.

Variety	Soluble Solids (%)	Polyphenols (g/L)	Flavonoids (%)	Protein (%)	Soluble Sugars	Score
YT93-159	21.00 ± 0.71 a	1.21 ± 0.00 a	0.58 ± 0.00 a	0.27 ± 0.03 a	13.08 ± 0.02 g	47.72
Dianzhe 01-104	16.67 ± 0.47 e	0.83 ± 0.05 b	0.03 ± 0.00 e	0.14 ± 0.01 cd	14.69 ± 0.01 d	9.30
Dianzhe 01-106	19.50 ± 0.41 c	0.71 ± 0.01 c	0.17 ± 0.00 b	0.18 ± 0.03 bc	14.35 ± 0.05 e	−0.04
Dianzhe 02-7	20.17 ± 0.24 b	0.26 ± 0.00 f	0.13 ± 0.03 c	0.21 ± 0.03 b	15.06 ± 0.01 c	−38.40
Dianzhe 09-38	18.83 ± 0.24 d	0.59 ± 0.01 d	0.16 ± 0.00 b	0.28 ± 0.02 a	15.48 ± 0.42 b	−14.66
Purple cane	15.00 ± 0.00 f	0.72 ± 0.01 c	0.05 ± 0.00 d	0.12 ± 0.03 d	13.68 ± 0.07 e	3.33
Yellow cane	19.00 ± 0.41 cd	0.45 ± 0.01 e	0.13 ± 0.00 c	0.14 ± 0.00 cd	15.98 ± 0.04 a	−27.17

Note: Values are means ± SD and data were extracted from [13]. Different lowercase letters within a column indicate significant differences at *p* < 0.05; the statistical procedures were those described in the original study [13].

**Table 3:** Sugarcane antioxidant activity indicators.

Variety	Total Polyphenols (mg/kg)	Total Flavonoid Content (mg/kg)	DPPH Radical Scavenging (%)	ABTS Radical Scavenging (%)	Total Reducing Power
YT93-159	878.22 ± 9.20 b	830.45 ± 2.01 c	83.02 ± 0.51 c	71.31 ± 0.16 c	1.16 ± 0.03 a
DZ09-78	554.25 ± 11.86 def	603.90 ± 7.66 f	58.39 ± 0.18 g	52.80 ± 0.68 d	0.72 ± 0.02 c
F134	585.70 ± 8.40 d	345.58 ± 6.03 i	67.37 ± 0.87 e	53.74 ± 0.37 d	1.15 ± 0.00 a
F172	1130.46 ± 30.73 a	1032.35 ± 19.39 a	94.38 ± 0.64 a	86.38 ± 0.58 a	1.18 ± 0.00 a
FN09-4095	532.62 ± 13.45 def	740.54 ± 13.26 d	46.73 ± 0.19 j	54.26 ± 0.51 d	0.56 ± 0.07 de
FN10-14405	528.63 ± 2.90 ef	690.99 ± 3.68 e	53.32 ± 0.36 h	40.78 ± 1.21 f	0.60 ± 0.01 d
FN43	520.09 ± 2.90 ef	468.77 ± 7.66 g	47.38 ± 0.38 j	36.78 ± 2.77 g	0.50 ± 0.01 ef
HT1	541.72 ± 25.95 def	459.76 ± 5.62 g	59.56 ± 0.69 f	53.84 ± 0.78 d	0.67 ± 0.01 c
LC05-136	564.57 ± 8.65 de	439.43 ± 0.00 g	44.73 ± 0.19 k	37.50 ± 0.89 g	0.44 ± 0.01 f
LC09-15	764.29 ± 14.65 c	857.66 ± 3.68 c	77.02 ± 0.81 d	70.74 ± 0.13 c	1.05 ± 0.10 b
XTT16	1123.85 ± 10.52 a	894.43 ± 5.32 b	87.16 ± 0.23 b	69.60 ± 0.33 c	1.07 ± 0.02 b
YZ08-1609	382.99 ± 7.64 g	385.40 ± 4.02 h	51.41 ± 0.13 i	39.69 ± 0.81 f	0.52 ± 0.00 def
ZT12-02	505.86 ± 1.61 e	677.48 ± 15.96 e	51.71 ± 0.36 i	47.26 ± 1.87 e	0.54 ± 0.01 de
ZT13-01	757.46 ± 4.90 c	836.64 ± 11.82 c	87.76 ± 0.10 b	77.10 ± 0.36 b	1.16 ± 0.11 a

Note: Values are means ± SD and data were extracted from [14]. Different lowercase letters within a column indicate significant differences at *p* < 0.05; the statistical procedures were those described in the original study [14].

**Table 4:** Breeding value parameters of YT93-159 and its representative cross combinations.

Trait/Parameter	YT93-159 as Male Parent	Guitang 02-467 × YT93-159	XTT22 × YT93-159	YT94-128 × YT93-159	Control (Mean of 55 Combinations)
GCA Effect (Sugar Content, %)	0.02	-	-	-	-
EBV	269.72	311.99	250.04	77.55	−29.86~411.59
SCA Effect (Sugar Content, %)	-	1.14	0.98	−1.36	−1.36~1.50
SCA Effect (Cane Yield, t/ha)	-	5.94	4.49	10.24	−14.56~17.31

Note: Data are extracted from [15]. GCA = general combining ability; SCA = special combining ability; EBV = economic breeding value. “-” indicates the parameter is not applicable for the corresponding category.

**Table 5:** Comparative summary of YT93-159 with mainstream improved sugarcane varieties in China based on published records.

Traits	YT93-159	GT42	GT44	LC05-136	YZ08-1609
Maturity type	Extra-early	Early and medium	Early	Early and medium	Early
Cane yield	102.42 t/ha (+13.99% vs. CK)	107.70 t/ha (+9.26% vs. CK)	107.85 t/ha (+5.20% vs. CK)	100.74 t/ha (+0.87% vs. CK)	105.50 t/ha (+14.40% vs. CK)
Sugar yield	16.73 t/ha, (27.07% vs. CK)	15.00 t/ha, (+14.45% vs. CK)	16.35 t/ha, (+12.82% vs. CK)	15.16 t/ha, (+5.41% vs. CK)	17.23 t/ha, (+22.30% vs. CK)
Key agronomic traits	Early sugar accumulation, wide adaptability, stable stress resistance	High tillering, early and fast growth, easy defoliation, wide adaptability	Strong ratooning, lodging resistance, mechanization suitability	Balanced yield–sugar performance, wide adaptability	Ultra-high sugar, strong continued sugar accumulation
Stress and disease resistance	DT: HR, CT: HR, SSDR: HR, SMDR: HR	DT: HR, SSDR: MR, SMDR: HR PBD: HR	DT: HR, SSDR: MR SMDR: HR	DT: HR, CT: HR, SSDR: MR, SMDR: MR	DT: HR SSDR: MS, SMDR: HR
Molecular feature	RNA-seq (disease response); sucrose/phenylpropanoid pathway gene regulation (candidate hypotheses)	Integrated transcriptomics–metabolomics; hormone-signaling and stress-response pathway activation	Integrated transcriptomics–metabolomics; same hormone/stress pathway features as GT42	Integrated transcriptomics–metabolomics; balanced yield–sugar gene–metabolite network	Integrated transcriptomics–metabolomics; ultra-high-sugar pathway regulation
Planting area in 2025	37,807 ha (3.45%)	235,080 ha (21.43%)	180,140 ha (16.42%)	265,160 ha (24.18%)	164,560 ha (15.00%)

Note: The comparisons are based on the published descriptions [7,8,9,10,16,17,45]; trial conditions, years, and check varieties differ among studies (GT42/GT44 vs. XTT22 in Guangxi regional trials; LC05-136 vs. XTT22 in national regional trials; YZ08-1609 vs. XTT22 in Yunnan regional trials; YT93-159 vs. XTT10 in Guangdong regional trials). LC05-136 has been found to be susceptible to sugarcane smut in recent years [9]. The planting area in 2025 was provided by the National Information Center for Sugarcane Industry. DT, drought tolerance; CT, cold tolerance; SSDR, sugarcane smut disease resistance; SMDR, sugarcane mosaic disease resistance; PBD, pokkah boeng disease; HR, highly resistant; MR, moderately resistant; MS, moderately susceptible.

## Data Availability

The data supporting the conclusions of this review are available in the manuscript, in the Supplementary Materials, and in the original publications cited. The raw RNA-seq data of sugarcane infected by smut were derived from the Genome Sequence Archive at the China National Center for Bioinformation (https://www.cncb.ac.cn/ (accessed on 26 October 2026)) under project number PRJCA011580 based on reference [16]. The baseline YT93-159-XTT22 comparison and the brown-rust time-course expression data were compiled from the authors’ earlier transcriptomic analyses reported in reference [17]; readers are referred to those studies for the primary data and analytical details. Regional-trial and variety-registration data are summarized in the official variety description and the cited statistical source for Table 1.

## Supplementary Materials

The following supporting information can be downloaded at: https://www.mdpi.com/article/10.3390/plants15213464/s1, Figure S1: The expression pattern of pivotal genes within ROS-scavenging genes; File S1: Transcriptomic expression levels of differentially expressed genes in diverse pathways between YT93-159 and the control variety (cited in Section 7).
